# Rectum-spacer related acute toxicity – endoscopy results of 403 prostate cancer patients after implantation of gel or balloon spacers

**DOI:** 10.1186/s13014-019-1248-6

**Published:** 2019-03-15

**Authors:** Andreas Schörghofer, Martin Drerup, Thomas Kunit, Lukas Lusuardi, Josef Holzinger, Josef Karner, Michael Groher, Christoph Zoubek, Rosemarie Forstner, Felix Sedlmayer, Frank Wolf

**Affiliations:** 1 Dapartment of Radiotherapy and Radio-Oncology, LKH Salzburg University Clinics, Paracelsus Medical University, Müllner Hauptstraße 48, 5020 Salzburg, Austria; 2 Department of Urology, LKH Salzburg University Clinics, Paracelsus Medical University, Müllner Hauptstraße 48, 5020 Salzburg, Austria; 3 Department of Radiology, LKH Salzburg University Clinics, Paracelsus Medical University, Müllner Hauptstraße 48, 5020 Salzburg, Austria; 4 Department of Surgery, LKH Salzburg University Clinics, Paracelsus Medical University, Müllner Hauptstraße 48, 5020 Salzburg, Austria

**Keywords:** Prostate, Spacer, Rectum toxicity, External beam radiation therapy

## Abstract

**Background:**

Rectal spacers are used to limit dose to the anterior rectal wall in high dose external beam radiation therapy of the prostate and have been shown to reduce radiation induced toxicity. Here we report the complication rate and toxicity of the implantation procedure in a large cohort of patients who have either received a gel- or balloon-type spacer.

**Methods:**

In total, 403 patients received rectal spacing, 264 with balloon, 139 with gel. Allocation was non-randomized. Two hundred seventy-six patients were treated with normofractionated regimen, the remaining 125 patients in moderate hypofractionation. Spacer related acute and late rectal toxicity was prospectively assessed by endoscopy using a mucosa scoring system (Vienna Rectoscopy Score) as well as CTCAE V.4. For the balloon subgroup, position and rotation of balloon spacers were additionally correlated to incidence and grade of rectal reactions in a post-hoc analysis of post-implant planning MRIs.

**Results:**

Overall rectal toxicity was very low with average VRS scores of 0.06 at the day after implantation, 0.10 at the end of RT, 0.31 at 6 months and 0.42 at 12 months follow up. Acute Grade 3 toxicity (rectum perforation and urethral damage) directly related to the implantation procedure occurred in 1.49% (*n* = 6) and was seen exclusively in patients who had received the spacer balloon. Analysis of post implant MR imaging did not identify abnormal or mal-rotated positions of this spacer to be a predictive factors for the occurrence of spacer related G3 toxicities.

**Conclusions:**

Spacer technology is an effective means to minimize dose to the anterior rectal wall. However, the benefits in terms of dose sparing need to be weighed against the low, but possible risks of complications such as rectum perforation.

## Introduction

For primary external beam radiotherapy (EBRT) of prostate cancer, dose escalation beyond 80Gy has become feasible with the advent of advanced radiation techniques such as intensity modulated radiation therapy (IMRT) and image guided radiation therapy (IGRT). However, the rectum remains to be a critical organ at risk (OAR) and the limiting factor for dose escalated treatments [1]. Even in the most conformal treatments such as combination treatment of advanced EBRT techniques with brachytherapy, rectal toxicity remains to be of concern.

The spatial separation of target structures and a given OAR is one of the simplest and most effective strategies to reduce dose to the anterior rectal wall. It can be achieved by placing a spacer material between rectum and prostate which is gradually absorbed after completion of therapy [2–4] (reviewed in [5]).

The most widely used spacer material is a polyethylene glycol gel (PEG) which polymerizes in seconds creating a hydrogel space (SpaceOAR™, Augmenix Inc., Waltham, MA). Following hydrodissection with a saline solution and confirmation of proper needle location by rectal ultrasound, the two liquid hydrogen precursors are injected into the perirectal space and then polymerize. SpaceOAR™ hydrogel was FDA-cleared in 2015. It has also received CE Mark approval in Europe, is approved in Australia and Japan, and is licensed in Canada.

Another approach is the implantation of a saline filled spacer balloon (ProSpace™, BioProtect Inc., Kfar-Saba, Israel) which is composed of biodegradable polymers. Once the balloon is in situ, it is inflated with sterile saline to reach its final configuration. The balloon remains inflated during the entire treatment period. CE Mark approval for ProSpace™ Balloon spacer was first issued in 2010. FDA approval is currently pursued by initiating a randomized multicenter study [6].

Both spacers biodegrade in the body within 3–6 months. Degradation products are absorbed and cleared via renal filtration.

Both approaches have been shown to effectively reduce dose to the anterior rectum wall in retrospective planning studies [2–4, 7]. Our group has previously demonstrated that the rectum surface encompassed by the 96% isodose was reduced by 35% for the gel, and by 63.4% by the balloon spacer at the time of planning [8]. This initial advantage of the balloon was somewhat mitigated due to a slow volume loss of 50% over a full treatment period of 8 weeks.

For the gel spacer, a clinical benefit has been demonstrated by a randomized study for acute as well as late toxicities [9, 10]. Hamstra et al. reported an improved 3-year incidence of grade ≥ 1 and ≥ 2 rectal toxicity as well as better bowel quality of life for the gel spacer group compared to patients who had not received a spacer.

Here we report spacer related toxicities inflicted by the implantation procedure in a large cohort of patients who have either received a gel or balloon spacer. In order to detect early mucosal defects which might be asymptomatic, we have performed rectal endoscopies for mucosal scoring using the *Vienna Rectoscopy Score* (VRS) [10] on a routine basis at the day after implantation as well as at the end of treatment and during follow up.

## Material and methods

### Implantation procedure

Balloon spacer ProSpace™: The implantation procedure is performed by an urologist under general anesthesia and has been described in detail in [7, 11]. In short, the balloon spacer is inserted with an applicator via a small perineal incision. Prior to insertion by means of a introducer kit, the retroprostatic space is dilated by hydrodissection. Subsequently the spacer balloon is placed and filled with 15-20 ml of physiological solution.

Hydrogel Spacer SpaceOAR™: Spacer application for SpaceOAR hydrogel has been described in detail in [12]. Hydrodissection is carried out with a 18Gx15cm needle. In contrast to the balloon spacer, no incision is needed as the spacer gel is injected via the same needle without the need of introducing an applicator.

### Rectal toxicity scoring

Rectal toxicity was assessed by performing endoscopy and scoring the rectal mucosa based on 5 domains using the VRS score as published ([13], see Table 1).

**Table 1:** Vienna Rectoscopy Score (VRS)

VRS	Congested mucosa	Telangiectasia	Ulceration	Stricture	Necrosis
Score 0	Grade 1	None	None	None	None
Score 1	Grade 2	Grade 1	None	None	None
Score 2	Grade 3	Grade 2	None	None	None
Score 3	Any	Grade 3	Grade 1	None	None
Score 4	Any	Any	Grade 2	Grade 1	None
Score 5	Any	Any	Grade ≥ 3	Grade ≥ 2	Any

Endoscopy and CTC scoring were performed at day 1 after implantation (timepoint (TP) 1), at the end of RT (TP2) 6 months after the end of RT (TP3) and 12 months after RT (TP4).

### Patient characterization and treatment delivery

From 11/2011 to 08/2017 403 patients with primary prostate cancer planned to receive definitive EBRT have been implanted with either spacer gel (SpaceOAR™, Augmenix Inc., Waltham, MA) or a rectal balloon (ProSpace™, BioProtect Inc., Kfar-Saba, Israel). Of note, the majority of patients received a balloon (*n* = 264); spacer selection was non-randomized and based on availability and at the discretion of the treating radiation oncologist. Patients of all risk groups were included (see Table 2 for distribution).

**Table 2:** Patient characteristics at baseline

	Number	
Age (mean)	75	(47–90)
Neoadjuvant hormonal therapy	145	(35.98%)
T stage		
T1a	3	(0.74%)
T1b	5	(1.24%)
T1c	186	(46.15%)
T2	102	(25.31%)
T2a	15	(3.72%)
T2b	16	(3.97%)
T2c	13	(3.23%)
T3	29	(7.20%)
T3a	1	(0.25%)
T3b	5	(1.24%)
T4	2	(0.50%)
PSA concentration (ng/mL)		
< 10	200	(49.63%)
10–20	127	(31.51%)
> 20	68	(16.87%)
Gleason score		
≤ 6	208	(51.61%)
7	127	(31.51%)
≥ 8	55	(13.65%)
Risk group		
Intermediate	170	(42.18%)
High	119	(29.53%)
Low	106	(26.30%)
Nadir	0.53	

Radiotherapy was administered using gold marker based IGRT and IMRT plans delivering either a normofractionated regimen of 75.85Gy in 41 fractions (68.49% of patients 49 + 21) or moderately hypofractionated fractionation regimens (31.02% of patients) with either 63Gy in 21fractions or 67.5Gy in 25 fractions (high risk patients only) (see Table 3). The average biologically equivalent dose (EQD2^αβ 1.5^) to the prostate based on an α/β ratio of 1.5Gy was 77.04Gy (range 75.85Gy–81Gy). In high risk patients, pelvic lymph nodes were treated up to 50Gy in 25 fractions. Dose-volume constraints for rectum and bladder were V70 < 20% and V60 < 35%, respectively, as recommended by QUANTEC [14].

**Table 3:** Treatment parameters

	Number	Percent
Normofractionation	276	68.49%
41 x 1.85Gy	276	68.49%
Hypofractionation	125	31.02%
21 x 3Gy	97	24.07%
25 x 2.7Gy	28	6.95%
Pelvic lymphnodes	116	28.78%

Androgen deprivation was routinely administered in intermediate risk (6 months) and high risk patients (24 months). Low risk patients did not receive hormonal therapy.

GM implantation, planning CT (3 mm slice thickness) and planning MRI were performed as described previously [8]. Prior to imaging, patients were instructed to have a full bladder and to empty their bowels using mild laxatives.

### Post-hoc analysis of spacer axis rotations

Using planning MR images which had been acquired after spacer implantation, the long axis of the balloon pacer was identified in transversal planes and its deviation from the anterior-posterior axis measured using the angle measurement tool of IMPAX EE R20 XV SU4 (AGFA HealthCare N.V.) software.

### Statistics

Spearman’s rho was computed with *SPSS 22* (IBM) for assessment of correlations. For all other calculations *Microsoft Excel 2007* was used.

## Results

### Rate of successful implantation

Of 494 patients planned to receive the spacer, the procedure was successfully performed in 403 patients (81.58%). 10.73% of patients did not receive clearance for anesthesia. In the remaining 7.69% of interventions, the procedure was not completed as intended due to valve deficiencies resulting in premature deflation (3.24%) or anatomic problems resulting in failure to hydrodissect the retroprostatic space (4.45%).

In 2015 the design of the plug mechanism has been optimized by Bioprotect. Since then, no deflation issues have occurred.

### Dose to organs at risk

Mean dose to the rectum and bladder was 24.71Gy (SD = 7.10) and 24.44Gy (SD = 12.70) for the balloon spacer and 29.92Gy (SD = 7.96) and 30.15Gy (SD = 12.68) for the gel spacer. The V50 for rectum and bladder was 8 and 18% for the balloon, and 16 and 23% for the gel spacer, respectively. A correlation of dose to the rectum and the CTC GI toxicity score was found (*r* = .201, *p* < .05).

### Vienna Rectoscopy scores

**Fig. 1:**
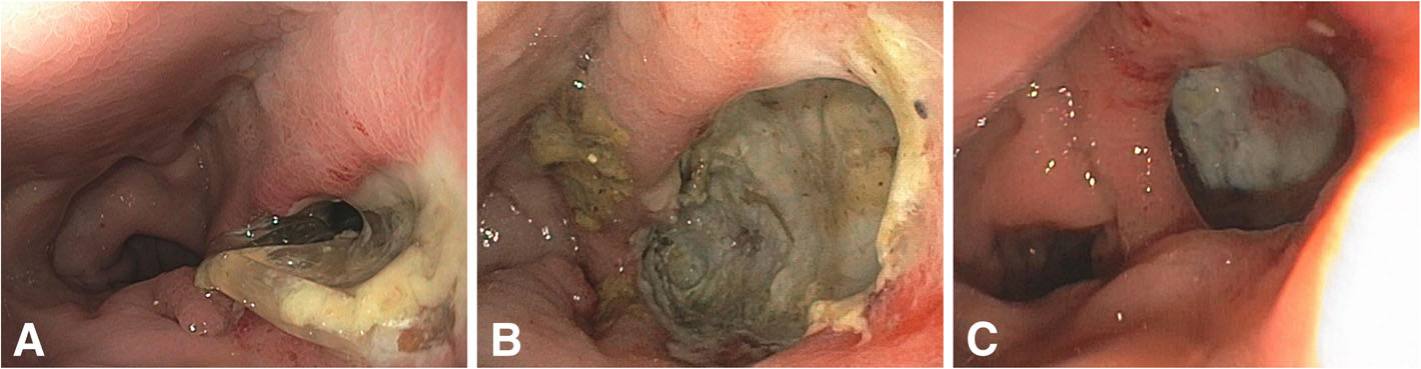
Rectum perforation: **a** initial. **b** 2 weeks. **c** 8 weeks

Overall rectal toxicity was very low with average VRS scores of 0.06 at the day after implantation (TP1), 0.10 at the end of RT (TP2), 0.31 at 6 months (TP3) and 0.42 at 12 months follow up (TP4) (see Table 4). At endoscopy performed at TP 1, 96.21% of all spacer patients presented with a VRS of 0. However five cases of rectum perforations were recorded in patients who had received the balloon spacer, corresponding to a temporary VRS score of 5 in 1.24% of all patients (1.89% of balloon patients). Of these, two were detected during routine endoscopy at the day after implantation. Following healing of the perforation both patients received full radiation treatment to 76Gy. One perforation was detected at day 30 after spacer implantation after the patient had complained about rectal pain. The radiation had to be stopped at 40Gy. The fourth and fifth rectum perforations were detected 98 and 108 days after implantation of the balloon spacer. At this time point the two patients had already finished the full treatment with a cumulative dose of 63Gy and 75.85Gy, respectively. All perforation sites healed without any complications or surgical intervention (see Fig. 1).

**Table 4:** Rectal toxicity as scored using VRS: VRS scores pretherapeutic (TP1), at the end of RT (TP2) and 6 (TP3) and 12 months follow up (TP4). Avg = average, S1 + 2 = summated score 1 & 2, S > 2 = score > 2

	VRS pre (TP1)			VRS end (TP2)			VRS post 6 (TP3)			VRS post 12 (TP4)		
	Avg	S1/2	S > 2	Avg	S1/2	S > 2	Avg	S1/2	S > 2	Avg	S1/2	S > 2
Gel	0.03	2.16%	0.00%	0.15	7.19%	0.72%	0.31	10.79%	0.72%	0.58	26.62%	0.00%
Balloon	0.09	1.89%	0.76%	0.05	1.52%	0.38%	0.32	5.30%	0.00%	0.26	6.06%	0.38%
Both	0.06	1.99%	0.50%	0.10	3.47%	0.50%	0.31	7.20%	0.25%	0.42	13.15%	0.25%

Of note, before the first patient had experienced a perforation, 59 balloon spacers had been implanted without any complications, suggesting that the learning curve of the implanting urologists does not seem to play a dominant role in the occurrence of complications.

### CTC scores

In addition to VRS scoring, toxicity was assessed using CTCAE v4.0 scoring for the following gastrointestinal and urogenital domains: hematuria, urinary frequency, incontinence, retention and urgency, Diarrhea, fecal incontinence, proctitis, rectal hemorrhage and rectal ulcer. For analysis, the highest grade of any gastrointestinal (GI) and any urogenital (GU) domain was scored.

Acute grade 1 and 2 GI toxicity was very low (TP1:1.99%; TP2: 10.92%). Acute grade 3 GI toxicity was limited to the five patients who had suffered a rectum perforation (1.24%).

Acute grade 1 and 2 urogenital toxicity was 20.84 and 30.27% at TP1 and TP2, respectively. Grade 3 GU toxicity was limited to one case of urethral laceration which was inflicted during the implantation of a balloon spacer. The patient suffering from urethral lazeration and fistula completed radiation but later underwent salvage radical cystoprostatectomy due to a persistent fistula.

Overall cumulated CTC grade 3 toxicity of any domain (GI and GU) at any timepoint was 1.24% (of all spacer patients (*n* = 403) and only occurred in patients who had received the balloon spacer (*n* = 264).

Late G1 and G2 toxicity at 6 and 12 months after radiation was 6.20 and 5.96% for GI and 26.31 and 23.82% for GU toxicity, respectively. No late G3 toxicity was recorded at any of the two timepoints.

### Spacer placement and radiologic findings

In order to identify factors which may be predictive for the development of spacer related complications such as rectal perforations, planning MRI datasets of balloon patients, which had been acquired at the day of the implantation, were analyzed for deviations of the spacer position (rotational and translational) as well as for the presence of a visible hematoma.

**Fig. 2:**
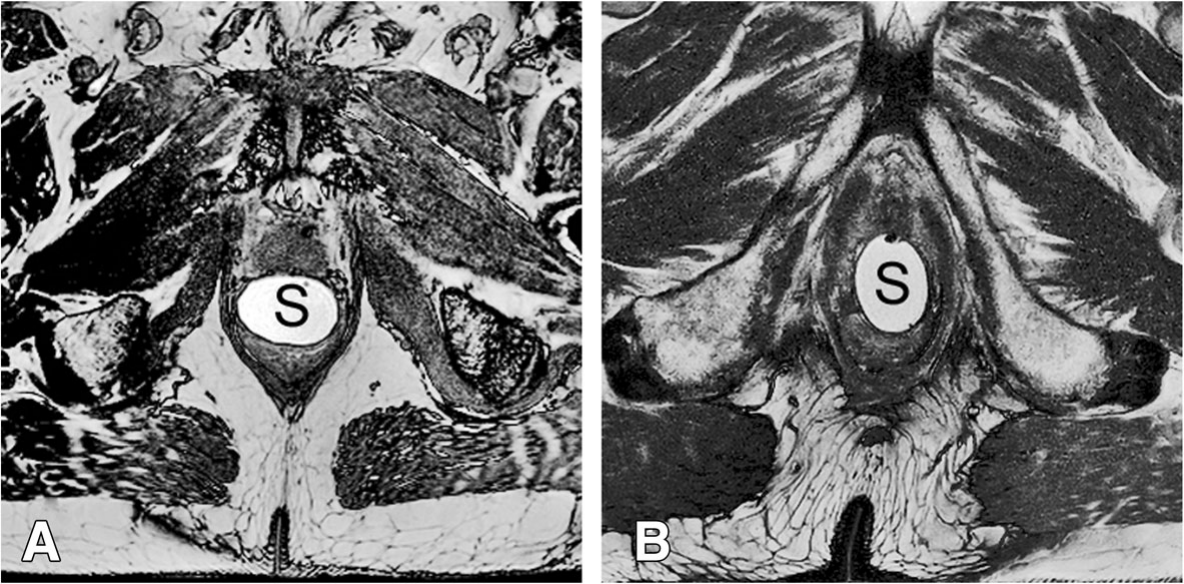
**a** Regular configuration of the balloon spacer with the long axis in left/right direction. **b** Rotated configuration with the long axis in anterior/posterior direction. S = balloon spacer

Mean rotation in the transverse plane of the balloon spacer was 24 degrees. In 10% of patients, the deviation was more than 64° (see Fig. 2).

Since a faulty spacer configuration may inflict increased pressure onto the rectum mucosa we correlated axis deviations to rectum toxicity. However, transverse axis deviations did not correlate with either increased VRS score (*p =* .361) or GI CTC score (*p =* .145) at any timepoint.

## Discussion

In our retrospective analysis we have analyzed a large cohort of patients who had either received a gel or balloon spacer for toxicities which are likely to be directly related to the spacer itself rather than to the radiation treatment. We therefore assessed rectal toxicity using endoscopy on the day after spacer implantation as well as at the end of RT and during follow up.

In terms of mild toxicities (grade 1 and 2) assessed at later timepoints it is impossible to discriminate between spacer related and radiation induced toxicities. However, any toxicity observed during the initial endoscopy (performed the day after implantation) as well as all reported grade 3 toxicities (5 rectum perforations and 1 urethral lazeration) were clearly directly related to the spacer placement itself. On a side note, it is important to point out that a CTC score of 3 in any of the gastrointestinal domains would correspond to a grade 4 toxicity in the old RTOG scoring system which was used in most of the dose escalation and hypofractionation trials.

It has clearly been shown that rectal spacers reduce acute as well as late grade 1 and ≥ 2 rectal toxicity [9]. In our cohort, overall rectal toxicity was also very low. Nonetheless, when looking at average toxicities scores, a decrease in grade 1 and 2 toxicities in many patients might be outbalanced by an increase of grade 3 toxicities induced by the spacer in some patients.

In our study we have shown that rectal spacers lead to an increased rate of grade 3 toxicities of 1.24–1.49%. Whether this can be accepted depends on the risk-benefit relation of the used fractionation scheme. When applied in modern IGRT-IMRT based technique, normo- as well as moderately hypofractionated regimens with biologically equivalent doses of approximately 78Gy are unlikely to cause any CTC grade 3 (corresponding to RTOG grade 4) or worse toxicities. ([15–19]). The benefit of a spacer application in such regimens is questionable since the reduction in radiation induced grade 1 and 2 toxicity may come at the price of increased grade 3 toxicity inflicted by the spacer. However, in highly dose escalated regimens with EQD2 of >80Gy and in stereotactic treatments where even higher BEDs are used, the benefit of the spacer becomes more likely to outweigh its risks. We therefore suggest that prior to implementing a spacer technique, a careful risk-benefit analysis should be carried out for the intended fractionation scheme. In addition, virtual spacers can be considered which allow to estimate the dose sparing benefit of a spacer prior to its actual implantation and therefor tailor the decision of a spacer implantation [20].

In our analysis, only patients who had received the balloon spacer experienced grade 3 toxicity. However, in the literature rectum perforations have also been reported for the gel spacer [21]. Spacer injection into the muscularis mucosae of the rectum wall may occur in both spacer types. However, for the gel, rectal wall infiltration has been shown to be tolerated without severe consequences [22]. In contrast, misplacement of the balloon spacer may be more prone to cause rectum perforation due to its rigid structure and size. In addition, the inflicted trauma during implantation is somewhat greater due to the larger applicator. Meanwhile, the implant procedure of the balloon spacer has been technically elaborated by the producer with the aim to reduce the risk of a possible spacer misplacement: hydro dissection is now carried out using a blunt dilator instead of a needle which is supposed to lower the risk of perforating the rectal wall. Future studies are required to verify these assumptions.

We have tried to identify possible causes of rectum perforations by looking at post implant MR images, but could not identify any predispositions such as mal-rotation or hematoma.

In our opinion, the most likely cause of rectum perforations was an unprecise placement of the needle in the retroprostatic space behind the Denovillier fascia prior to hydrodissection. Such a faulty needle position might be facilitated by adhesions e.g. caused by chronic prostatitis. If true, blunt hydrodissection may be an effective improvement to avoid this.

Limitations to the study are the following: This is a non-randomized observational study without a control group and the number of patients in the gel group (*n* = 139) and the balloon group (*n* = 264) is not evenly distributed, which in part might explain the higher perforation rate in this cohort. Comorbidities which might have been predisposing for bleeding such as diabetes mellitus, vascular diseases etc. were not investigated for balanced distribution between the groups. Moreover, in total four different (although experienced) urologists performed the respective application maneuvers, possibly contributing to bias.

To fully address late toxicities, 12 months follow up is too short. However, since both the gel and the balloon spacer are dissolved and resorbed after 6 to 12 months, the advent of spacer related late toxicities after that time is very unlikely and we do not think that a longer follow up will provide additional useful information.

Nonetheless, and to the best of our knowledge, the present paper reports the largest single-institution experience with rectal spacers regarding acute and subacute toxicity.

## Conclusion

A rectal spacer technology is a safe and effective method to minimize dose to the anterior rectal wall. However, although the overall complication rate is below 2%, the benefits of spacer technologies need to be weighed against the possible risks of complications such as rectum perforation/necrosis. For standard as well as moderately hypofractionated regimens at EQDs up to 78Gy we therefore recommend to prioritize other non-invasive dose sparing methods such as IGRT and IMRT over spacer technology. However, rectum spacers can be an important asset if higher-dose escalated (>80Gy) or extremely hypofractionated treatment regimens (stereotactic body radiation therapy, SBRT) are to be established.

## Abbreviations

CT: Computer tomography
CTCAE V.4: Common toxicity criteria, version 4.0
EBRT: External beam radiotherapy
EQD2: Equivalent dose in 2Gy per fraction
GI: Gastrointestinal
GM: Gold marker
GU: Urogenital
IGRT: Image guided radiation therapy
IMRT: Intensity modulated radiation therapy
MRI: Magnet resonance imaging
OAR: Organ at risk
PEG: Polyethylene-glycol gel
RT: Radiation therapy
TP: Time point
VRS: Vienna Rectoscopy Score
